# Triple Space‐Polarization On–Off‐Key Modulation With AlN‐Based Tri‐Focal Metalens

**DOI:** 10.1002/nap2.70266

**Published:** 2026-09-18

**Authors:** Zhi‐Yan Lin, Chen‐Yi Tsai, Bo‐Yi Chen, Huang‐Yu Wei, Hsiao‐Hsuan Yang, Yuh‐Jen Cheng, Hideto Miyake, Chia‐Yen Huang

**Affiliations:** ^1^ Department of Photonics National Yang Ming Chiao Tung University Hsinchu Taiwan; ^2^ Research Center for Applied Sciences Academia Sinica Taipei Taiwan; ^3^ Department of Electrical and Electronic Engineering Mie University Mie Japan

## Abstract

We designed and demonstrated a tri‐focal metalens (TF‐lens) made of high‐temperature‐annealed AlN (HT‐AlN) on sapphire. The HTA‐AlN film possesses an ultrawide bandgap (*E*_*g*_ = 6.2 eV), atomic‐level smoothness, and single‐crystalline quality, making it an exceptional candidate for a metasurface in visible spectral wavelengths. By incorporating polarization‐insensitive zone‐plate null rings into a polarization‐selective bifocal metalens, the incident light will be focused into two or three foci on the focal plane, each with a distinct polarization state. The extinction ratio between the on and off peaks exceeds 1000 after subtracting the background signal, and the absolute value of *S*_3_ is measured to be higher than 0.96 at the side foci. By manipulating the incident polarization state and the angle of the linear analyzer behind the TF‐lens, all combinations of the on–off keying at three distinct foci can be generated.

## Graphical abstract

An AlN tri‐focal metalens made of high‐temperature‐annealed AlN by integrating zone‐plate null rings into a polarization‐selective bifocal metalens. The incident light will be focused into two or three foci on the focal plane, each with a distinct polarization state. By attaching polarization optics, all combinations of the on–off keying at three distinct foci can be generated.

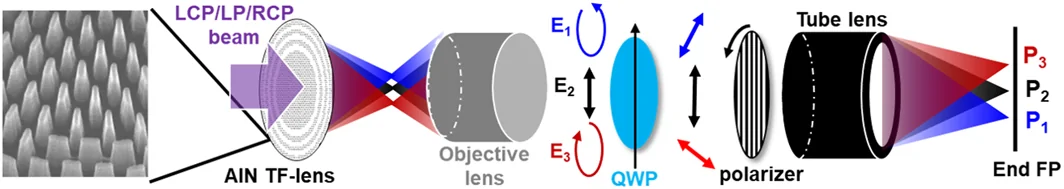

## 1 Introduction

Compound semiconductors are strong candidates for metasurfaces due to their high refractive index and high transparency in the subbandgap spectral region. A compound semiconductor‐based metasurface can also be monolithically integrated with active optoelectronic devices made of the same materials, such as light‐emitting diodes [1, 2, 3, 4, 5] and surface‐emitting lasers (SEL) [6, 7, 8, 9, 10, 11], to modulate the out‐coupling beam within an ultra‐compact space. For example, the beam from the SELs can be split into two beams with orthogonal polarizations by modulating the Pancharatnam–Berry (geometric) phase of birefringent meta‐atoms [12]. A particular optical spin can be extracted from a linearly polarized light by meta‐atoms with mixed phases [13, 14]. A beam with orbital angular momentum (OAM) can also be generated by imposing a helical phase profile encoded in the metasurface [15, 16, 17]. The generation of optical spin and OAM enhances the information density of photons with the same wavelength, which is critical for next‐generation information technologies, such as quantum information [18, 19, 20] and visible‐light communications [21]. Among all the compound semiconductors, AlN has the widest wavelength compatibility for metasurfaces due to its ultrawide bandgap (*E*_*g*_ = 6.2 eV) and a sufficiently high refractive index. Robust and efficient AlN metalenses have recently been demonstrated from 266 to 375 nm [22, 23, 24]. However, the polycrystalline nature of the conventional sputtered AlN film imposed practical challenges in the fabrication of AlN metasurfaces. As the dimensions of the meta‐atom are close to the grain size of the AlN nano‐column, fabricating the nano‐pillar with high pattern fidelity via dry etching becomes critical. Therefore, a homogeneous AlN film is strongly desired for sophisticated metasurfaces. Over the past decade, atomically flat and nearly single‐crystalline high‐temperature‐annealed AlN (HTA‐AlN) films on sapphire substrates have been developed as a growth template for efficient deep‐UV light‐emitting diodes [25, 26, 27]. These HTA‐AlN films are also an ideal platform for metasurfaces in the UV–visible spectral range. Figure 1a,b show the optical constants and surface morphology of the HTA‐AlN film used in this study. The refractive indices are above 2.2 from 200 to 700 nm, and the transmittance is above 50% down to 210 nm. The bi‐layer atomic‐terrace morphologies implied exceptional surface flatness and thickness homogeneity, which GaN on sapphire heteroepitaxy could not achieve at submicrometer thicknesses. The high film quality in submicrometer thickness is important for the meta‐atom's pattern fidelity. Because the sapphire substrate serves as the natural etch‐stop layer for III‐nitrides, the etching time window for meta‐atom fabrication is broader if the meta‐atom height matches the initial film thickness. As long as the meta‐atom library can cover 2π phases and has a decent transmittance, a lower pillar height is preferred from a fabrication perspective. Therefore, the thinner HTA‐AlN buffer could be a more favorable choice for a metasurface than GaN heteroepitaxy in the visible spectral region, even though GaN might have a higher refractive index.

**FIGURE 1:**
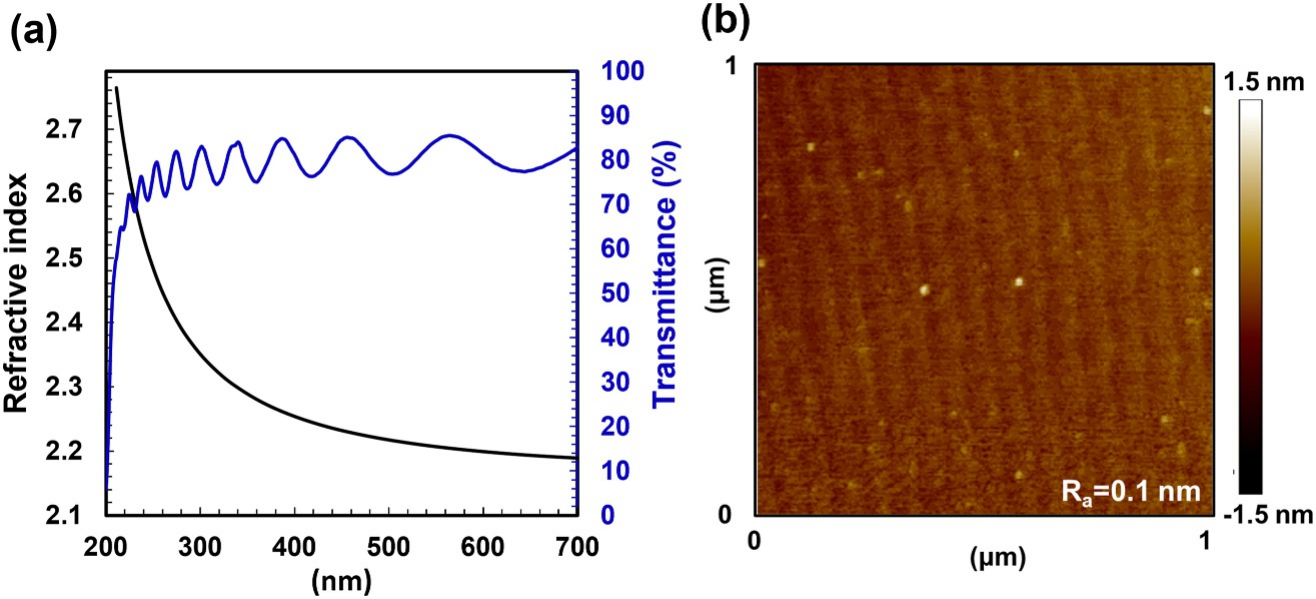
(a) The index dispersion and transmittance curve and (b) the 1 μm × 1 μm atomic‐force microscopy image of an HTA‐AlN on sapphire.

In this work, we design and fabricate a tri‐focal metalens (TF‐lens) with HTA‐AlN as shown in Figure 1. By integrating the polarization‐sensitive bifocal lens (BF‐lens) and the polarization‐insensitive zone plate (ZP), the incident beam is split and focused into three space divisions with different polarization states. The on and off states at each division can be modulated by controlling the polarization of the incoming and outgoing beams using external polarization optics. The TF‐lens holds strong potential to enhance the capacity of applications that rely on the binary information transmitted by photons.

## 2 Design and Simulation

### 2.1 Construction of the TF‐Lens

To independently manipulate photons with opposite spin states using a single array of densely packed meta‐atoms, both the propagation phase and geometric phase of polarization‐sensitive meta‐atoms must be engineered to fully exploit the available degrees of freedom. A schematic of the AlN rectangular nanofin structure is presented in Figure 2a. A hexagonal lattice is employed due to its higher filling factor, which helps mitigate efficiency degradation associated with the Nyquist limit. The lattice period is set to 360 nm, and the nanofin height is 600 nm. The Jones matrix presentation of light passing through the nanofin with the basis of the unit left‐circularly polarized (LCP) and right‐circularly polarized (RCP) spins is written as Equation (1) [13, 23]:

(1) $$\left[\begin{array}{c} E_{L,\text{out}}\\ E_{R,\text{out}} \end{array}\right]=\left[\begin{array}{cc} \frac{T_{o}e^{i\varphi_{o}}+T_{e}e^{i\varphi_{e}}}{2} & \frac{T_{o}e^{i\varphi_{o}}-T_{e}e^{i\varphi_{e}}}{2}e^{-i2\theta }\\ \frac{T_{o}e^{i\varphi_{o}}-T_{e}e^{i\varphi_{e}}}{2}e^{i2\theta } & \frac{T_{o}e^{i\varphi_{o}}+T_{e}e^{i\varphi_{e}}}{2} \end{array}\right]\left[\begin{array}{c} E_{L,\text{in}}\\ E_{R,\text{in}} \end{array}\right],$$

where θ is the rotation angle between the reference axes and the principal axes of the ordinary ray and the extraordinary ray. The parameters $T_{o}$ and $T_{e}$ represent the real‐valued electric‐field transmissivity, whereas $\varphi_{o}$ and $\varphi_{e}$ denote the associated propagation phases of the o‐ray and e‐ray, respectively. The $T_{o}$/$T_{e}$ and $\varphi_{o}$/$\varphi_{e}$ of a nanofin with given $W_{x}$ and $W_{y}$ are calculated with Reticolo‐RCWA by assuming the polarization of the plane wave to be along the reference *x*‐axis. To further simplify the matrix formulation, we introduce the root‐mean‐square transmissivity ($T_{\text{rms}}$) and the half‐wave plate factor ($\eta_{\text{HWP}}$), as defined in Equations (2) and (3):

(2) $$T_{\text{rms}}=\sqrt{\frac{T_{e}^{2}+T_{o}^{2}}{2}},$$

(3) $$\eta_{\text{HWP}}^{2}=\frac{{2T}_{c}^{2}}{T_{o}^{2}+T_{e}^{2}}=\frac{1}{2}-\frac{T_{o}T_{e}}{T_{o}^{2}+T_{e}^{2}}\;\cos\left(\varphi_{o}-\varphi_{e}\right).$$

**FIGURE 2:**
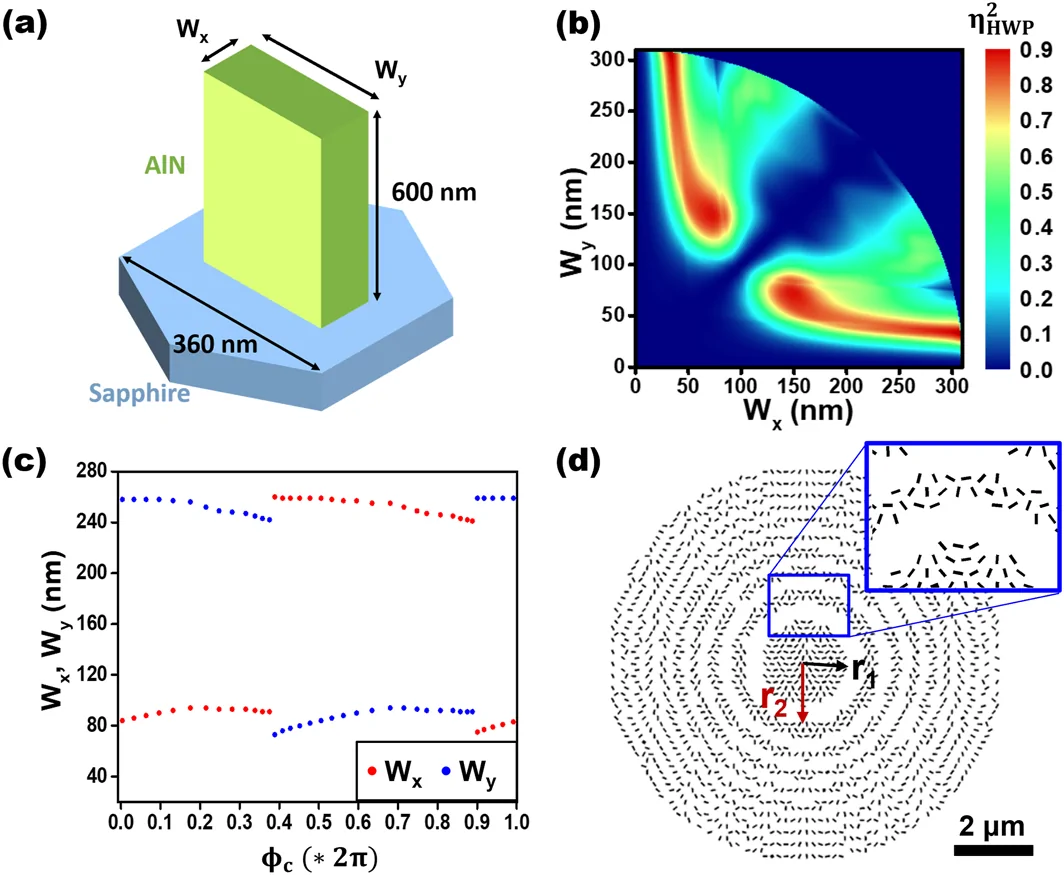
(a) The schematic structure of the AlN meta‐atom, (b) the color mapping of $\eta_{\text{HWP}}^{2}$ with *W*_*x*_ and *W*_*y*_, (c) the look‐up table of *W*_*x*_ and *W*_*y*_ for an optimized $\eta_{\text{HWP}}^{2}$ value, and (d) schematic TF‐lens with *F* = 16 μm and a diameter of 10 μm.

Substituting these into Equation (1) yields Equation (4):

(4) $$\left[\begin{array}{c} E_{L,\text{out}}\\ E_{R,\text{out}} \end{array}\right]=T_{\text{rms}}\left[\begin{array}{cc} \sqrt{1-\eta_{\text{HWP}}^{2}}e^{{i\varphi }_{u}} & \eta_{\text{HWP}}e^{i\left(\varphi_{c}-2\theta \right)}\\ \eta_{\text{HWP}}e^{i\left(\varphi_{c}+2\theta \right)} & \sqrt{1-\eta_{\text{HWP}}^{2}}e^{{i\varphi }_{u}} \end{array}\right]\left[\begin{array}{c} E_{L,\text{in}}\\ E_{R,\text{in}} \end{array}\right],$$

$\phi_{u}$ and $\phi_{c}$ are the effective propagation phases of the unconverted and converted spins, given by Equations (5) and (6):

(5) $$\varphi_{u}=\mathrm{Arg}\left(T_{o}e^{i\varphi_{o}}+T_{e}e^{i\varphi_{e}}\right),$$

(6) $$\varphi_{c}=\mathrm{Arg}\left(T_{o}e^{i\varphi_{o}}-T_{e}e^{i\varphi_{e}}\right).$$

If the metasurface is constructed with meta‐atoms with $\eta_{\text{HWP}}\;∼1$, that is, the HWP ensemble, Equation (4) reduces to Equation (7):

(7) $$\left[\begin{array}{c} E_{L,\text{out}}\\ E_{R,\text{out}} \end{array}\right]=T_{\text{rms}}\left[\begin{array}{c} E_{R,\text{in}}e^{i\left(\varphi_{c}-2\theta \right)}\\ E_{L,\text{in}}e^{i\left(\varphi_{c}+2\theta \right)} \end{array}\right]=T_{\text{rms}}\left[\begin{array}{c} E_{R,\text{in}}e^{i\varphi_{R}}\\ E_{L,\text{in}}e^{i\varphi_{L}} \end{array}\right].$$

According to Equation (7), the LCP component of an arbitrary incident beam sees $\varphi_{L}=\varphi_{c}+2\theta$, and the RCP component sees $\varphi_{R}=\varphi_{c}-2\theta$; then, the polarization switches to the opposite spin. As long as the phase $\varphi_{c}$ of the HWP ensemble spans a full 2π range, the $\phi_{L}$ and $\phi_{R}$ can be independently assigned. Figure 2b maps the calculated $\eta_{\text{HWP}}^{2}$ as a function of *W*_*x*_/*W*_*y*_ in Figure 2a. After filtering those with $\eta_{\text{HWP}}^{2}$ > 0.99, $\phi_{c}$ as a function of W_x_/*W*_*y*_ is plotted in Figure 2c as the look‐up table for metasurface construction. The geometric parameters *W*_*x*_ and *W*_*y*_ are determined from Figure 2c based on a target phase $\varphi_{c}=\left(\varphi_{L}+\varphi_{R}\right)∕2$.

Equations (8) and (9) are the assigned metalens profiles for the RCP and LCP components, respectively. The focal length (F) is identical, but the foci are spatially displaced along the x‐axis from the origin:

(8) $$\varphi_{R}\left(x,y\right)=\frac{2\pi }{\lambda }\left(F-\sqrt{F^{2}+{\left(x-x_{0}\right)}^{2}+y^{2}}\right),$$

(9) $$\varphi_{L}\left(x,y\right)=\frac{2\pi }{\lambda }\left(F-\sqrt{F^{2}+{\left(x+x_{0}\right)}^{2}+y^{2}}\right),$$

where *F* = 16 μm and *x*_0_ = 3 μm for the following simulations.

The third focus is introduced by incorporating the zone plate into the bifocal metalens. The radius of each ring is defined by Equation (10):

(10) $$r_{n}=\sqrt{n\lambda F-\frac{1}{4}n^{2}\lambda^{2}}.$$

The regions with $r_{2m-2}<r<r_{2m-1}$ correspond to the *m*th ring of the bifocal metalens, where meta‐atoms are arranged accordingly; those with $r_{2m-1}<r<r_{2m}$ are the *m*th ring of the binary zone plate, which is left blank. With retrieved *W*_*x*_/*W*_*y*_ from Figure 2c, and $\theta =\left(\phi_{L}-\phi_{R}\right)∕4$, and ZP, a TF‐lens pattern with a diameter of 10 μm can be generated, as shown in Figure 2d.

### 2.2 Response to the Incident Polarization

The generated metalens structures were imported into Ansys Lumerical for verification using the finite‐difference time‐domain (FDTD) method. The light was configured to propagate along the +*z*‐axis from the substrate toward the air. The incident beams were set as plane waves originating from the sapphire substrate, with a wavelength of 450 nm, whereas the polarization was varied among linear polarization along the *y* direction (LP‐y), RCP, and LCP. The focal plane intensity distributions and their corresponding profiles along the *x*‐axis (*y* = 0) are shown in Figure 3. Under LP‐y incidence (Figure 3b), three intensity peaks—*P*_1_, *P*_2_, and *P*_3_—emerge. As LP‐y can be viewed as a superposition of LCP and RCP, this result clearly demonstrates the combination of spin‐dependent splitting by the bifocal lens (P_1_ and P_3_) and zero‐order focusing of the ZP (*P*_2_). The polarization of *P*_1_/*P*_3_ will be in the RCP/LCP state, whereas that of *P*_2_ maintains its original polarization state. As shown in the intensity profile of Figure 3d–f, the inherent intensity ratio of the side peaks (*I*_*s*_) to the central peak (*I*_*c*_) is approximately 0.77 under LP‐y incidence, whereas that under pure RCP or LCP incidence is doubled. The full‐width at half‐maximum (FWHM) of the intensity peak and the *I*_*s*_/*I*_*c*_ ratios are summarized in Table 1.

**FIGURE 3:**
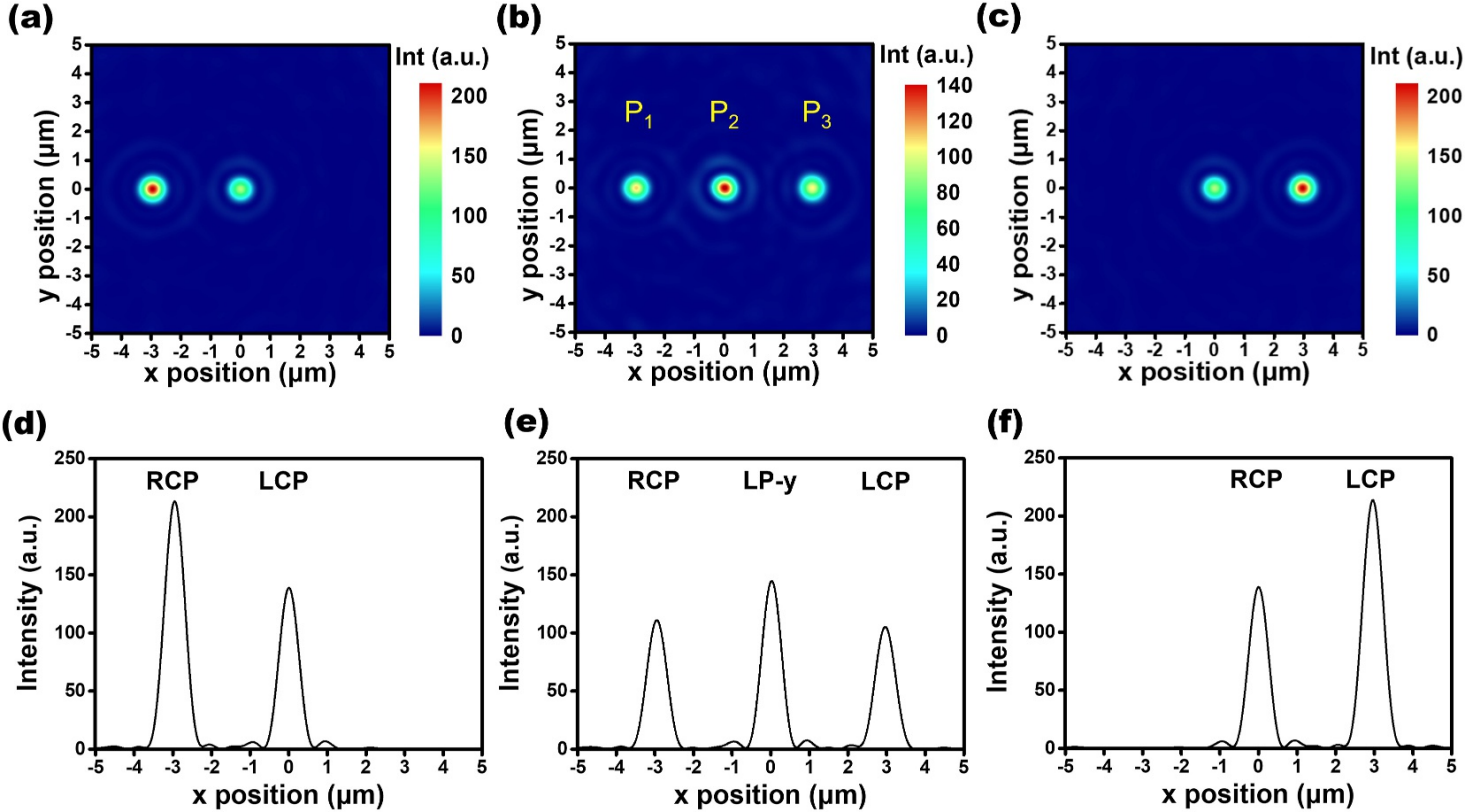
The intensity mapping on the focal plane by FDTD simulation with an incident polarization of (a) LCP, (b) LP‐y, and (c) RCP. The corresponding intensity profiles across three foci are plotted in (d)–(f) below. The polarization states of each peak are marked above.

**TABLE 1:** Summary of simulated and experimental peak FWHMs and *I*_*s*_/*I*_*c*_ under LCP/RCP or LP‐y incidence on the TF‐lens.

	*I*_*s*_ FWHM (nm)	*I*_*c*_ FWHM (nm)	*I*_*s*_/*I*_*c*_ under LCP/RCP	*I*_*s*_/*I*_*c*_ under LP‐y (= *α*)
Simulation	800	641	1.54	0.77
Experiment	855	651	1.42	0.71

The focusing efficiencies at the side points ($\eta_{s}$) and the central points ($\eta_{c}$) are calculated by dividing the integrated intensity near the focal points by the integrated intensity transmitted through the metasurface. Under an LP‐y incidence, $\eta_{s}$ = 4.7% for each side focus and $\eta_{c}$ = 6.4% for the central focus. The total focusing efficiency, η, under LP‐y incidence is evaluated to be 15.8%. Of the optical energy loss, 52.1% is attributed to the energy leakage through high‐order diffractions and 32.1% is attributed to the background straying optical energy in the focal plane. The definition and calculation of the above efficiencies can be found in Supporting Information S1.

## 3 Results and Discussion

### 3.1 Experimental Verification

An HTA‐AlN buffer layer was deposited on a 2‐inch (001) flat sapphire substrate by sputtering and then atomically smoothed by MOCVD regrowth. The AlN total thickness is estimated to be ∼600 nm by the ellipsometer. The backside of the sapphire substrate was polished to a mirror‐like surface. To facilitate experimental alignment, the metalens diameter (250 μm), focal length F (400 μm), and displacement $x_{0}$ (30 μm) of the TF‐lens were scaled up by a factor of 25 compared to the FDTD simulations, while maintaining a constant numerical aperture (NA ≈ 0.3).

The fabrication process began with the deposition of a 300‐nm SiO_2_ layer on the AlN template using plasma‐enhanced chemical vapor deposition (PECVD). Subsequently, a 100‐nm layer of poly(methyl methacrylate) (PMMA‐A3) was spin‐coated as a positive‐tone resist for electron beam lithography (EBL). After development, rectangular apertures were formed in the PMMA layer. A 30‐nm Cr layer was then deposited via e‐gun evaporation, followed by a lift‐off process in acetone to transfer the pattern. The pattern was subsequently transferred from the Cr hard mask to the SiO_2_ layer using a CHF_3_/Ar plasma mixture in a reactive ion etching (RIE) system. Finally, the structures were etched into the AlN layer using a Cl_2_/BCl_3_/Ar gas chemistry. Figure 4a–c presents scanning electron microscopy (SEM) images of the fabricated TF‐lens at various magnifications and positions. Due to the hard mask's limited selectivity, the sidewalls exhibit a smooth profile but are slightly tapered from the ideal vertical orientation.

**FIGURE 4:**
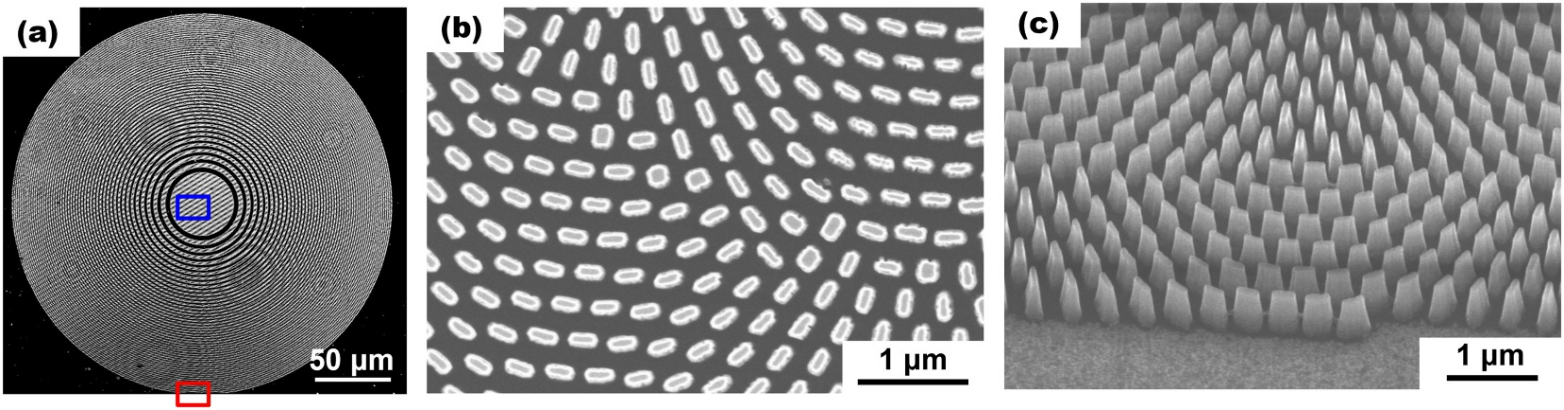
(a) The SEM top‐view image of the fabricated TF‐lens. (b) The top view of the blue rectangular area and (c) the bird view of the red rectangular area in (a) under higher magnification.

The schematic measurement setup is shown in Figure 5a. The incident light source was a 450‐nm TEM_00_ diode laser system. The incident beam was in LP‐y state with a divergence angle of < 0.3 mrad. The beam size was first reduced using a 300‐μm‐diameter iris, and the polarization state was subsequently modulated by a rotational quarter‐wave plate (QWP, Thorlabs). The metalens was mounted on a homemade 3D‐printed fixture and aligned perpendicular to the optical axis. A near‐ultraviolet transparent 50× infinity‐corrected objective lens (Mitutoyo, f=200μm), together with a tube lens and a charge‐coupled device (CCD, Thorlabs), was mounted on a motorized linear translation stage that moves along the optical axis. The TF‐lens was optically aligned so that its focal plane coincided with that of the 50× objective lens, thereby enabling a magnified image of the near‐field intensity distribution on the CCD. The focal position was identified at the point where the intensity profile reached its minimum width. In addition, a tunable neutral density (ND) filter placed before the iris was used to prevent signal saturation on the CCD. The CCD images with different incident polarizations are shown in Figure 5b. The measured focal length is approximately 60μm, which is in excellent agreement with the design value. The corresponding intensity profile is shown in Figure 5c. To evaluate the polarization state at each focus, the optical energy is collected by a single‐mode fiber that connects to a commercial polarimeter (ThorLabs). The measured *S*_3_ values at *P*_1_, *P*_2,_ and *P*_3_ under LP‐y incidence are 0.98, 0.02, and −0.96, respectively, which are in good agreement with the theoretical calculations, 0.98, 0, and −0.98.

**FIGURE 5:**
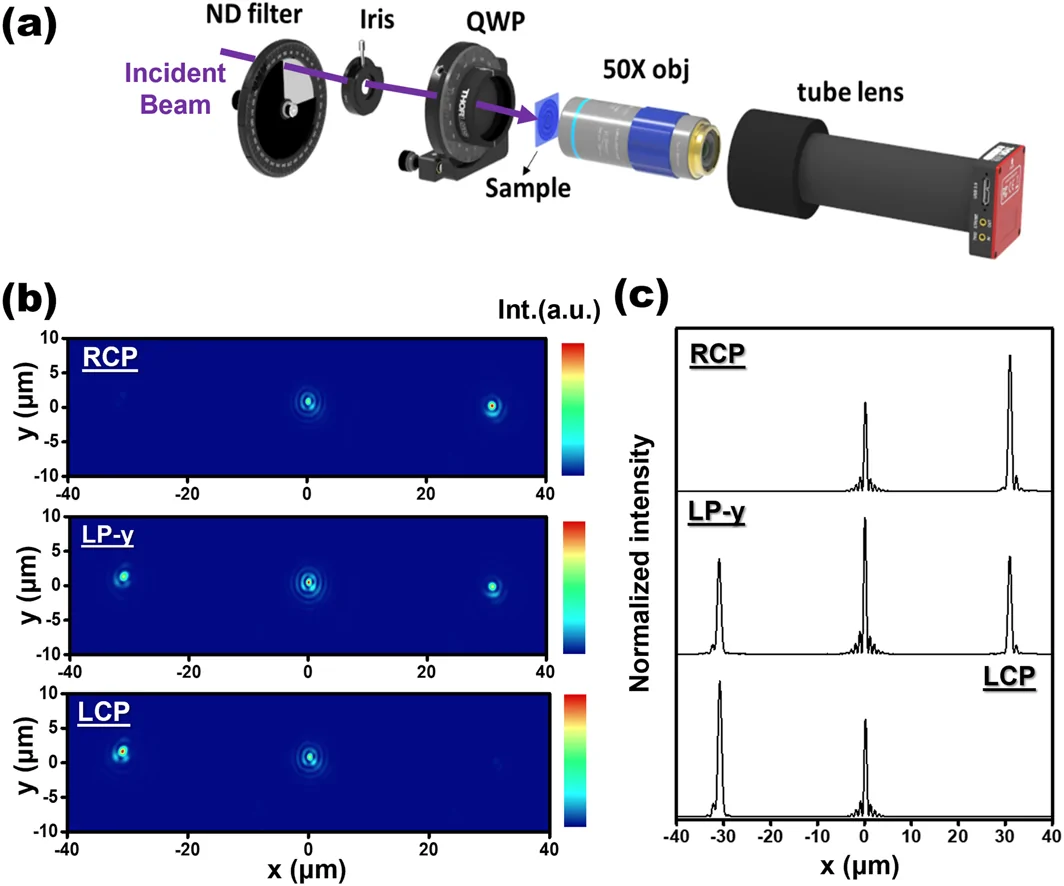
(a) The schematic setup for intensity measurement on the focal plane. (b) Measured intensity profile with different incident polarization states marked at the corner. (c) The intensity profile across three foci.

The experimental and simulated FWHMs and the *I*_*s*_/*I*_*c*_ are summarized in Table 1. The measured FWHM values at the side and central focal points are approximately 855 and 651 nm, respectively, which are in close agreement with the simulated predictions (800 and 641 nm) obtained under the same NA. Furthermore, the measured *I*_*s*_/*I*_*c*_ values closely match simulations under LCP, RCP, and LP‐y incidence conditions. The extinction ratio between the “on” and “off” states in Figure 5c is > 1000 after the background noise subtraction, implying negligible optical crosstalk for practical applications. Notably, the *I*_*s*_/*I*_*c*_ ratios in Table 1 are natural outcomes of the TF‐lens following the construction rules in Section 2.1, and those values could change with a different given NA. There might be a target *I*_*s*_/*I*_*c*_ ratio of the TF‐lens for some applications. In the following sections, we will discuss the targeted ratio and how it can be actively achieved regardless of the given NA.

### 3.2 Triple Space‐Division On–Off Keying (3SD‐OOK)

In many applications, such as photolithography and data communication, photons carry only binary information: on (= 1) and off (= 0). If the TF‐lens can be used to transmit on and off states at three different points, the information density per shot is tripled by spatial‐division multiplexing. To implement all possible combinations of the triple‐spatial‐division on–off keying (OOK), a quarter‐wave plate (QWP2) and a linear polarizer (LP) are integrated behind the objective lens, as illustrated in Figure 6a. By leveraging Malus's law, the relative intensity between the focal points can be precisely modulated by attaching polarization optics to achieve seven distinct OOK states as summarized in Table 2.

**FIGURE 6:**
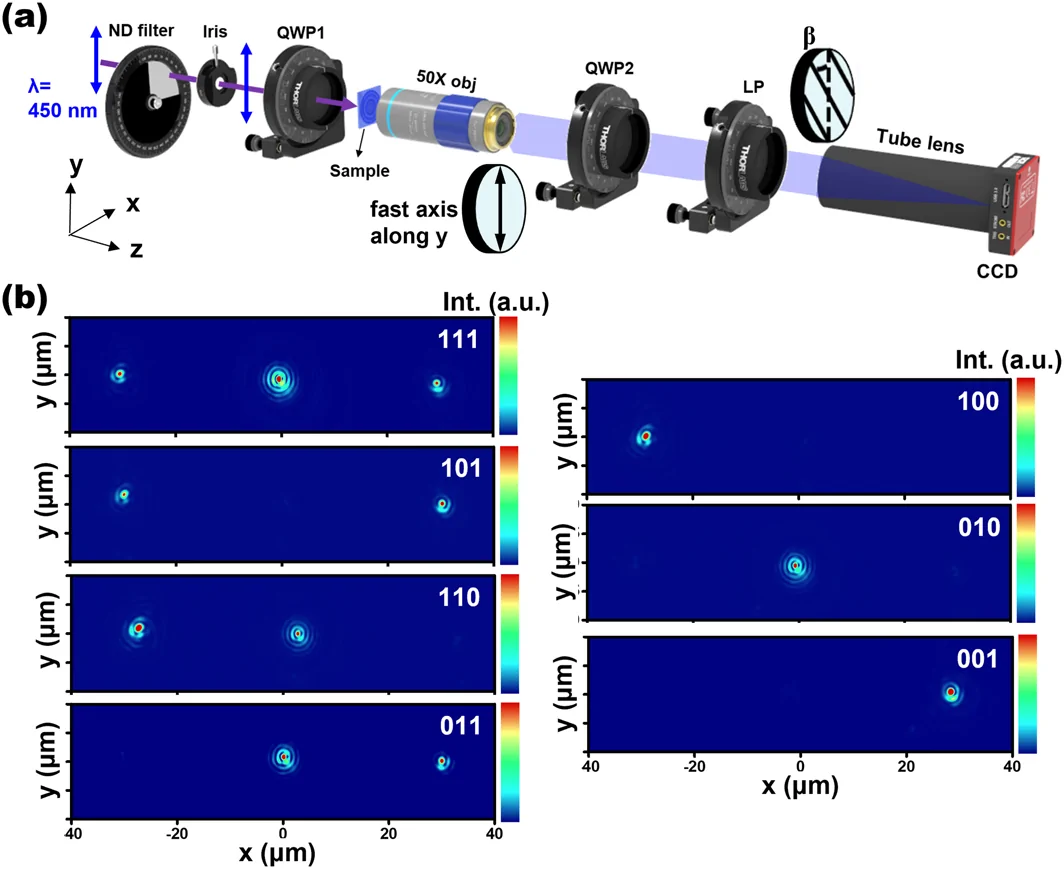
(a) The schematic setup for the on–off keying generation at each focus and (b) the measured intensity profile on the final focal plane based on the OOK state marked at the corner and configuration listed in Table 2.

**TABLE 2:** Summary of the QWP1 and LP configurations and intensity ratios for all OOK states except the 000.

Polarization state			*I*_1_:*I*_2_:*I*_3_ w/o LP	*β* of LP	*I*_1_:*I*_2_:*I*_3_ w/LP	OOK state
After QWP1	w/o QWP2	w/QWP2				
LP‐y	$E_{1}∥\hat{R}$ $E_{2}∥\hat{y}$ $E_{3}∥\hat{L}$	$E_{1}∥\hat{x}+\hat{y}$ $E_{2}∥\hat{y}$ $E_{3}∥\hat{x}-\hat{y}$	*α*:1:*α*	0	*α*/2:1:*α*/2	111
				π/2	1:0:1	101
LCP	$E_{1}∥\hat{R}$ $E_{2}∥\hat{L}$	$E_{1}∥\hat{x}+\hat{y}$ $E_{2}∥\hat{x}-\hat{y}$	2*α*:1:0	π/4	1:0:0	100
				$\beta^{\prime }$	1:1:0	110
				−π/4	0:1:0	010
RCP	$E_{2}∥\hat{R}$ $E_{3}∥\hat{L}$	$E_{2}∥\hat{x}+\hat{y}$ $E_{3}∥\hat{x}-\hat{y}$	0:1:2*α*	$-\beta^{\prime }$	0:1:1	011
				−π/4	−π/4	001

*Note*: 0 = off, 1 = on.

In the first configuration, the fast axis of QWP1 is aligned with the LP‐y incident light to preserve its original polarization state as it enters the TF‐lens, thereby forming three focal points. After magnification by the objective lens, the light passes through QWP2 (fixed with its fast axis along the *y*‐axis), which converts the circular polarization of the foci into linear states along the $\hat{x}\pm \hat{y}$ direction. Assuming *I*_*s*_/*I*_*c*_ to be *α* under LP‐y incidence, the intensity ratio among three foci after passing through the LP at β=0 becomes *α*/2:1:*α*/2 by Malus's law. Therefore, if we want to transmit the 111 state with uniform intensity across three points, *α* must be 2. In this report, the *α* of the fabricated device is 0.71, as noted in Table 1. The strategy of actively tuning *α* will be discussed later in Section 3.3. If the LP is rotated to β=π/2, the central channel is blocked, thereby generating the 101 OOK state.

Subsequently, if the TF‐lens is under pure circularly polarized incidence (LCP and RCP), the TF‐lens generates only two foci. The pristine intensity ratios without LP will be 2*α*:1:0 or 0:1:2*α*. Because these configurations involve only two active foci with orthogonal polarization states, the 100, 010, and 001 OOK states can be selectively controlled by setting β=±π/4 to allow only a single beam to pass. For the 110 and 011 states, the required β to equalize the intensities of the two “on” states can also be derived via Malus's law, yielding $\beta =\pm \beta^{\prime }=\pm \left[\frac{\pi }{4}-\mathrm{atan}\left(\sqrt{\frac{1}{2\alpha }}\right)\right]$. Finally, the 000 state is achieved by turning off the source. The experimentally captured CCD images of the seven OOK states, following the configurations in Table 2, are shown in Figure 6b. Notably, the absolute peak intensities among the seven combinations in Table 2 are not necessarily equal under the same incident power due to the conservation of energy. If the application requires uniform peak intensity across seven OOK states, an additional input‐power control unit is required.

### 3.3 Potential Applications

#### 3.3.1 TF‐Lens as a Generalized Polarization Splitter

From Table 1, *I*_*s*_/*I*_*c*_ under LP‐y incidence is around α= 0.71. Considering a unit y‐polarized electric field, $E_{\text{in}}$ = $\hat{y}$, field is incident; the electric field at the focal plane, E_FP_, can be written as in Equation (11):

(11) $$E_{\text{FP}}\left(\hat{x}\right)=E_{0}\left(\sqrt{\alpha }\hat{R}{\dot{P}}_{1}+\hat{y}{\dot{P}}_{2}+\sqrt{\alpha }\hat{L}{\dot{P}}_{3}\right),$$

where *E*_0_ is the normalizing amplitude, $\hat{L}$ and $\hat{R}$ are the unit LCP and RCP electric fields, and ${\dot{P}}_{i}$ presents the *i*th focal point position. For an incident beam with an arbitrary polarization, $E_{\text{in}}$, the E_FP_ can be generalized to Equation (12):

(12) $$E_{\text{FP}}\left(E_{\text{in}}\right)=E_{0}\left[\sqrt{2\alpha }\langle \hat{L},E_{\text{in}}\rangle \hat{R}{\dot{P}}_{1}+E_{\text{in}}{\dot{P}}_{2}+\sqrt{2\alpha }\langle \hat{R},E_{\text{in}}\rangle \hat{L}{\dot{P}}_{3}\right],$$

where $\langle \hat{L},E_{\text{in}}\rangle$ is the inner product of E_in_ and $\hat{L}$, same for $\langle \hat{R},E_{\text{in}}\rangle$. If the electric field at each focus is collected by different optical fibers, the TF‐metalens serves as a generalized polarization beam splitter, extracting the LCP and RCP components from *P*_1_ and *P*_3_, respectively, while simultaneously keeping part of the original beam from P_2_. The *α* can be intentionally designed over a wide range, as depicted in Figure 7. If we fill the ZP rings with the meta‐atoms of the BF‐lens design, *α* increases with the BF‐lens's weight. On the other hand, if part of the BF‐lens meta‐atoms are replaced with polarization‐insensitive meta‐atoms with a π‐phase shift, *α* decreases with the increasing ZP's weight.

**FIGURE 7:**
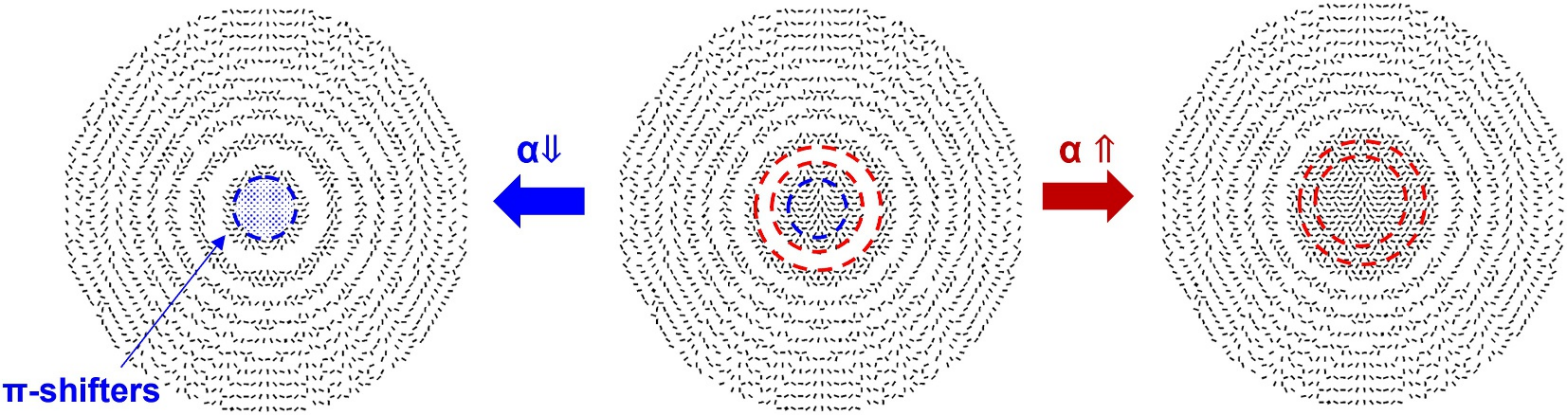
Schematic representation of tuning *α* by partial replacement of BF‐lens with π‐shifters (left) or gap‐filling with BF meta‐atoms (right).

The TF‐lens could be used to extract the relative phase of coherent photons, an important parameter in quantum optics [28, 29, 30]. For example, considering ideal photons under the Bloch sphere presentation, $\psi \rangle =\cos\;\theta |\hat{L}\rangle +\sin\;\theta e^{i\phi }|\hat{R}\rangle$ incident on a TF‐lens, we can analyze its relative phase, ϕ, by measuring the *x*‐polarized component intensities on the focal plane. The *x*‐component intensity at the *P*_*i*_, *I*_*ix*_, can be calculated by substituting ψ〉 into Equation (12), yielding Equations ((13), (14), (15)):

(13) $$I_{1x}=\alpha E_{0}^{2}\;\cos^{2}\;\theta ,$$

(14) $$I_{2x}=E_{0}^{2}\left(1+\sin\;2\theta \;\cos\;\phi \right)/2,$$

(15) $$I_{3x}={\alpha E}_{0}^{2}\;\sin^{2}\;\theta .$$

Then, θ and ϕ can be evaluated from the measured *I*_*ix*_ with a precalibrated α. The derivation of Equations ((13), (14), (15)) and the extraction of θ and ϕ can be found in Supporting Information S1.

#### 3.3.2 Compatibility With Other Materials Platforms

Beam‐splitting metalenses have been demonstrated across various material platforms, and the objectives varied from the wavelength [31] and polarization states [23, 32, 33] to the topological charge of optical vortices [34, 35]. Some representative works are summarized in Table 3. For metalenses built on the simultaneous modulation of geometric and propagation phases (i.e., the hybrid phases), the number of foci is limited to 2 for a single‐phase profile due to the limited degrees of freedom. One could also increase the number of foci by imposing multiple‐phase profiles on the same plane by meta‐atom sampling or tiling the area with different metalenses. We refer to these approaches as spatial multiplexing. In this work, embedding concentric null rings can be considered a derivative of spatial multiplexing. However, our approach only removes meta‐atoms rather than replacing them with different ones. Therefore, it shall be more broadly compatible with existing hybrid‐phase‐based bifocal metalenses across other material platforms, thereby further enhancing their functionality.

**TABLE 3:** Summary of the beam‐splitting multifocal metalenses on the forming materials, number of foci, objectives, and approaches.

Materials	Number of foci	Objective	Approach	Ref.
AlN	3	Polarization	Hybrid phases + zone plate	This work
AlN	2	Polarization	Hybrid phases	[23]
Ag	2	Polarization	Spatial multiplexing	[32]
Liquid crystal	2	Polarization	Hybrid phases	[33]
TiO_2_	3	Wavelength	Spatial multiplexing	[31]
TiO_2_	4	Topological charge	Spatial multiplexing	[34]
TiO_2_	2 × l	Topological charge	Azimuthal quadratic phases	[35]

## 4 Conclusion

In conclusion, we designed and fabricated a tri‐focal metalens that integrates a polarization‐insensitive zone plate and a polarization‐sensitive bifocal metalens. The distinctive polarization states at three foci enable all possible combinations of the on and off states in each space division with a single laser pulse. We believe that the unique polarization response of the tri‐focal metalens will enhance capacity and functionality in many applications that rely on transmitting binary information with photons.

## Author Contributions

**Zhi‐Yan Lin:** investigation, methodology, validation. **Chen‐Yi Tsai:** writing – original draft, formal analysis. **Bo‐Yi Chen:** writing – review and editing. **Huang‐Yu Wei:** visualization. **Hsiao‐Hsuan Yang:** software, visualization. **Yuh‐Jen Cheng:** visualization, supervision. **Hideto Miyake:** resources. **Chia‐Yen Huang:** conceptualization, writing – original draft, writing – review and editing, project administration, methodology.

## Conflicts of Interest

The authors declare no conflicts of interest.

## Supporting information


Supporting Information S1

## Data Availability

The data that support the findings of this study are available from the corresponding author upon reasonable request.
