# Supplementary material for: Triple Space‐Polarization On–Off‐Key Modulation With AlN‐Based Tri‐Focal Metalens
Source: Nanophotonics. 2026 Sep 18;15(18):e70266. doi: 10.1002/nap2.70266 (PMC13672130; doi:10.1002/nap2.70266)
Supplement: Supplementary file 1 [file NAP2-15-e70266-s001.docx]

Triple space-polarization on-off-key modulation with AlN-based tri-focal meta-lens

Zhi-Yan Lin^1^, Chen-Yi Tsai^1^, Bo-Yi Chen^1^, Hsiao-Hsuan Yang^1^, Hideto Miyake^2^, Chia-Yen Huang^1^

1. Department of Photonics, National Yang Ming Chiao Tung University, No.1001 Ta Hsueh Rd, Hsinchu, 30010, Taiwan

2. Department of Electrical and Electronic Engineering, Mie University, Mie 514-8507, Japan

E-mail: [cyhuang06@nycu.edu.tw](mailto:cyhuang06@nycu.edu.tw)

**I. Diffraction efficiency (**$\boldsymbol{\eta}_{\boldsymbol{d}}$**) and focusing efficiency (η) of the tri-focal metalens:**

To calculate the diffraction efficiency and focusing efficiency, the a 20 μm-wide TF-lens is imported into Ansys Lumerical. The metalens is placed on the horizontal x-y plane as in Figure S1(a) and a 450 nm plane wave is injected from the substrate side toward the +z direction. The sidewalls are set to be perfectly matched layers (PML), and three monitor planes were included. The first monitor plane is the horizontal xy-plane above the metasurface, z=z_0_; the second is the horizontal plane passing through the three focal points, z=z_f_; the third is the vertical xz-plane passing through the three focal points, y=0. The intensity profile on the y=0 plane is plotted in Figure S1(b). The local intensity peaks below the z=z_f_ are due to the higher-order diffraction. Here we define $\eta_{d}$ as the ratio of energy arriving at the focal plane to the initial integrated intensity. $\eta_{d}$ is calculated by equation (S1):

$$\eta_{d}=\frac{\int_{|r|\leq r_{0}} I(x,y)dxdy|_{z=z_{f}}}{\int_{|r|\leq r_{0}} I(x,y)dxdy|_{z=z_{0}}} (S1)$$

r is the in-plane position vector to the center metalens, and r_0_ is the radius of the metalens (= 10 μm). $\eta_{d}$calculated is calculated to be 47.9%. It implies 52.1% of the optical energy is leaving the optical axis and thereby be absorbed by the PML through the high-order diffractions.

We further calculate the focusing efficiency of each focal point using equations (S2) and (S3):

$$\eta_{s}=\frac{\int_{|r-P_{1}|\leq{\Delta r}_{s}} I(x,y)dxdy|_{z=z_{f}}}{\int_{|r|\leq r_{0}} I(x,y)dxdy|_{z=z_{0}}} (S2)$$

$$\eta_{c}=\frac{\int_{|r-P_{2}|\leq{\Delta r}_{c}} I(x,y)dxdy|_{z=z_{f}}}{\int_{|r|\leq r_{0}} I(x,y)dxdy|_{z=z_{0}}} (S3)$$

$\eta_{s}$ and $\eta_{c}$ is the focusing efficiency around the side points (P_1_ and P_3_) and center point (P_2_) in Figure S1(b), Δr_s_ and Δr_c_ is 1.5 times of the intensity FWHM of each focal spot, respectively. Equations (S2) and (S3) only integrate the optical energy in the vicinity of each focal point in the numerator. Assuming an LP-y incidence, $\eta_{s}$ and $\eta_{c}$ are estimated to be 4.7% and 6.4% respectively. The total focusing efficiency ($\eta$) can be calculated by equation (S4):

$$\eta={2\eta}_{s}+\eta_{c} (S4)$$

, which is 15.8%. The difference between $\eta$ and η_d_ indicates the stray optical energy that the TF-lens could not focus on the designed focal plane.

Figure S1 (a) Schematic of the FDTD simulation with designed monitor planes. (b) The intensity mapping in the y=0 monitor plane.

**II. Extracting the relative phase of coherent photons with TF-lens:**

From Section 3.3.1, the electric field at the focal plane, **E_FP_**, with a given incident polarization state, **E_in_**, is written as Equation (S5):

$E_{FP}\left( E_{in} \right)=E_{0}\left[ \sqrt{2\alpha}\left\langle\hat{L},E_{in} \right\rangle\hat{R}\dot{P}_{1}+E_{in}\dot{P}_{2}+\sqrt{2\alpha}\left\langle\hat{R}, E_{in} \right\rangle\hat{L}\dot{P}_{3} \right]$…(S5)

After substituting $E_{in}=cos\theta|\left. \hat{L} \right\rangle+sin\theta e^{i\phi}|\left. \hat{R} \right\rangle$ into (S5), equation (S6) yields:

$E_{FP}\left( E_{in} \right)=E_{0}\left[ \sqrt{2\alpha}cos\theta\hat{R}\dot{P}_{1}+(cos\theta\hat{L}+sin\theta e^{i\phi}\hat{R})\dot{P}_{2}+\sqrt{2\alpha}sin\theta e^{i\phi}\hat{L}\dot{P}_{3} \right]$	(S6)

If the x-polarized component of **E_FP_** is extracted by a linear polarizer, equation (S6) becomes equation (S7):

$E_{FP}\left( E_{in} \right)=E_{0}\left[ \sqrt{\alpha}cos\theta\hat{x}\dot{P}_{1}+\frac{1}{\sqrt{2}}(cos\theta+sin\theta e^{i\phi})\hat{x}\dot{P}_{2}+\sqrt{\alpha}sin\theta e^{i\phi}\hat{x}\dot{P}_{3} \right]$	(S7)

The intensity profile at the focal plane is evaluated by $I_{FP}=E_{FP}^{*}E_{FP}$, yielding equation (S8):

$I_{FP}=E_{0}^{2}\left[ \alpha{cos}^{2}\theta\dot{P}_{1}+\frac{1}{2}(1+sin 2\theta cos\phi)\dot{P}_{2}+\alpha{sin}^{2}\theta\dot{P}_{3} \right]$	(S8)

If the measured intensity at $\dot{P}_{i}$ is I_i_, we have the equations (S9) to (S11) by comparing with equation (S8).

$I_{1}=\alpha E_{0}^{2}{cos}^{2}\theta$	(S9)

$I_{2}=E_{0}^{2}(1+sin 2\theta cos\phi)$/2	(S10)

$I_{3}={\alpha E}_{0}^{2}{sin}^{2}\theta$	(S11)

The $cos\theta$ and $cos\phi$ can be isolated by algebraic operations as in equations (S12) and (S13):

$$cos\theta=\sqrt{\frac{I_{1}}{I_{1}+I_{3}}} (S12)$$

$$cos\phi=\frac{\alpha I_{2}-(I_{1}+I_{3})/2}{\sqrt{I_{1}I_{3}}} (S13)$$

Thus, $\theta$ and $\phi$ can be evaluated from measured I_i_’s with a pre-calibrated $\alpha$.
